# Deploying Acoustic Detection Algorithms on Low-Cost, Open-Source Acoustic Sensors for Environmental Monitoring

**DOI:** 10.3390/s19030553

**Published:** 2019-01-29

**Authors:** Peter Prince, Andrew Hill, Evelyn Piña Covarrubias, Patrick Doncaster, Jake L. Snaddon, Alex Rogers

**Affiliations:** 1 School of Electronics and Computer Science, University of Southampton, Southampton SO17 1BJ, UK; ah1u14@soton.ac.uk; 2 School of Biological Sciences, University of Southampton, Southampton SO17 1BJ, UK; e.pinacovarrubias@soton.ac.uk (E.P.C.); cpd@soton.ac.uk (P.D.); 3 School of Geography and Environmental Science, University of Southampton, Southampton SO17 1BJ, UK; j.l.snaddon@soton.ac.uk; 4 Department of Computer Science, University of Oxford, Oxford OX1 2JD, UK; alex.rogers@cs.ox.ac.uk

**Keywords:** acoustics, bioacoustics, ecology, conservation, machine learning

## Abstract

Conservation researchers require low-cost access to acoustic monitoring technology. However, affordable tools are often constrained to short-term studies due to high energy consumption and limited storage. To enable long-term monitoring, energy and space efficiency must be improved on such tools. This paper describes the development and deployment of three acoustic detection algorithms that reduce the power and storage requirements of acoustic monitoring on affordable, open-source hardware. The algorithms aim to detect bat echolocation, to search for evidence of an endangered cicada species, and also to collect evidence of poaching in a protected nature reserve. The algorithms are designed to run on AudioMoth: a low-cost, open-source acoustic monitoring device, developed by the authors and widely adopted by the conservation community. Each algorithm addresses a detection task of increasing complexity, implementing extra analytical steps to account for environmental conditions such as wind, analysing samples multiple times to prevent missed events, and incorporating a hidden Markov model for sample classification in both the time and frequency domain. For each algorithm, we report on real-world deployments carried out with partner organisations and also benchmark the hidden Markov model against a convolutional neural network, a deep-learning technique commonly used for acoustics. The deployments demonstrate how acoustic detection algorithms extend the use of low-cost, open-source hardware and facilitate a new avenue for conservation researchers to perform large-scale monitoring.

## 1. Introduction

Real-time analysis of data from acoustic sensors is increasingly becoming commonplace, with many research projects using low-cost micro-controllers and field-programmable gate arrays (FPGAs) to broadcast acoustic data to a central hub for analysis. This technique has found a wide range of applications, from monitoring noise pollution in urban environments [1] to non-intrusively monitoring threatened bird species [2]. To reduce the cost and energy requirements of environmental sensors, processing is now often carried out on the micro-controllers themselves, utilising their limited processing power to implement detection algorithms which recognise the unique vocalisations of many species [3,4].

There is increasing interest in the use of dedicated, power-efficient acoustic sensors that conservation researchers can use for large-scale, long-term monitoring of natural resources and anthropogenic impacts on the environment (such as poaching) [5]. The research in this area includes the development of self-powered sensors that use energy harvesting techniques such as solar panels [6] and triboelectric nanogenerators [7]. These charging methods are constrained by the amount of power they can produce in their operating environments, which may present suboptimal conditions for power generation. For example, where sensors are deployed under forest canopies (e.g. for conservation monitoring), they are likely to be shaded from direct sunlight, and lack the constant sound pressure required for triboelectric nanogenerators [8].

To further increase the utility of dedicated acoustic sensors, it is desirable that they implement detection algorithms such that instead of recording at fixed intervals, the devices are set to record in response to specific sounds [9,10]. By doing so, the energy consumption of the device is reduced significantly, as the act of writing an audio file to a device’s storage is often its most power-consuming task. Detection algorithms can also reduce storage requirements, as fewer recordings are made over the course of a deployment. In addition, reduced storage and battery costs result in lower overall costs for large-scale, long-term monitoring projects.

Currently, the majority of research projects in ecology and conservation using acoustics report using commercial devices such as the Wildlife Acoustics Song Meter series. Such devices achieve high sound fidelity at the cost of a high purchase price (US $800–1000 per unit) [11]. The high prices reflect the high quality of their microphones and the relatively small commercial market that they serve. However, not all applications require such fidelity. In these cases, the costs limit the scope and impact of the research, as well as their adoption in developing countries. Commercial monitoring devices are also closed-source, which makes it difficult or impossible to adapt their hardware or software to a specific application.

In response to the need for tailored acoustic monitoring devices, there is a growing trend in the research community to develop and use self-made devices [12,13]. In the past, environmental researchers without extensive electronics training have been hesitant to pursue home-grown tools due to the difficulty of building them from the ground up. However, recent modular micro-computers have provided a platform on which to build devices optimised for their application. These include the Raspberry Pi, which has formed the basis of a number of self-made monitoring devices [14,15,16,17]. The Raspberry Pi lends itself to this application since it is easy to expand, program, and configure. However, it typically runs a full Linux operating system, and thus is also relatively power-hungry, with consumption varying from 80 mA to 260 mA depending on the model and use. Long-term deployments consequently require higher-capacity power sources such as 12-volt automotive batteries, constraining the ability of these devices to provide substantial coverage on limited budgets [18].

**Figure 1:**
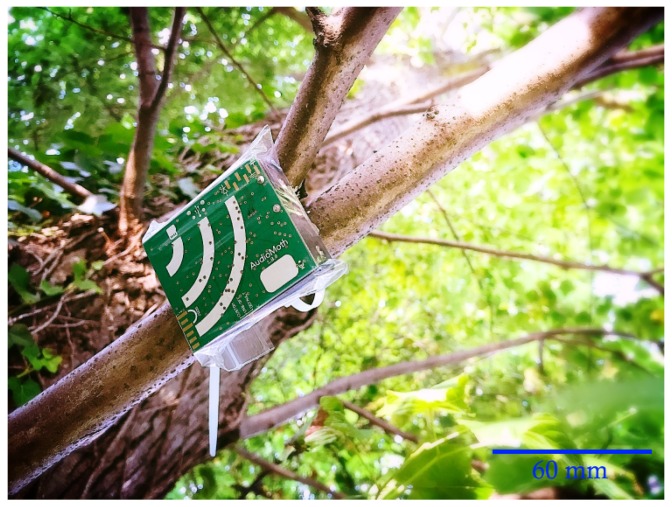
AudioMoth: a low-cost, low-power acoustic monitoring device developed for a wide variety of conservation projects, deployed on a tree in a grip-sealed bag using a cable tie.

To address these shortcomings, and to meet the need for such an open, flexible platform for acoustic monitoring applications, we have developed AudioMoth [19], a low-cost, open-source acoustic monitoring device (see Figure 1). The hardware and firmware are all open-source, with permissive licenses that allow AudioMoth to be modified and extended for any application without constraints (https://www.openacousticdevices.info/license). AudioMoth is the first open-source acoustic logger to be widely adopted by the conservation community. Due to its simple construction and open-source design, it can be manufactured cheaply through group funding. To date, since its launch in September 2017, over 3700 AudioMoth devices have been manufactured and deployed by conservation groups, researchers, and NGOs worldwide.

In this paper, we present a full technical evaluation of three acoustic detection algorithms which use the AudioMoth platform for applications that aim to monitor the environment and protect biodiversity. We describe the development process for these algorithms; each algorithm addresses a detection task of different complexity while operating within the computational restraints of the AudioMoth device. These tasks include analysing each sample multiple times to reduce the chance of missed events (for bat echolocation call detection), implementing additional analytical steps to account for environmental conditions such as wind (for the detection of the New Forest cicada), and performing classification using a hidden Markov model (HMM) to incorporate both the time and frequency domain of the sound (for gunshot detection). In order to fully benchmark the performance of the HMM, the same dataset was used to train a convolutional neural network. The storage constraints of the AudioMoth meant it was not possible to run the algorithm directly on the device. Instead, performance was compared to the HMM on desktop hardware.

We demonstrate achievement of the goal of low-cost environmental monitoring through the development of these algorithms, having specifically designed them to run on low-power, open-source hardware. Taken together, these algorithms demonstrate the utility of on-board acoustic detection when the application and its requirements result in constraints such as limited computational power, memory availability, and computation time. By making the device, its firmware, and the algorithms open-source, we hope to encourage the use of further acoustic detection algorithms for environmental monitoring, taking advantage of the low cost and flexibility of the AudioMoth platform.

The remainder of this paper is organised as follows: In Section 2 we briefly describe the AudioMoth, on which all detection algorithms were run. In Section 3 we describe the development of the acoustic monitoring algorithms, as well as the evaluation of performance of each in terms of its *F*${}_{1}$ score, precision, and recall accuracy metrics. Each algorithm had an accompanying trial which informed the design. In Section 3.5 the third algorithm is compared to an equivalent detection method making use of deep-learning techniques which have become commonplace within the field of acoustic analysis. Finally, we conclude with a discussion in Section 4 along with our plans for the continuation of various AudioMoth projects.

## 2. Design of the AudioMoth

### 2.1. Hardware

AudioMoth has been designed for a wide variety of applications. It captures sound through an analogue MEMS (microelectromechanical) microphone and an analogue preamplifier, supporting sample rates up to 384 kHz to give the device a broad spectrum frequency response from 20 Hz to 192 kHz. AudioMoth records audio as 16-bit uncompressed waveform audio (WAV) files to a microSD card. A back-up high precision real-time clock keeps track of each recording event, and can run in standby mode, using just 22 μA. The device weighs under 80 g and measures 58 × 48 × 18 mm, making it discreet and well-suited to mobile applications as well as static deployments [19].

AudioMoth is designed around an Arm Cortex-M4F processor operating at 48 MHz with hardware-enabled floating point arithmetic and 256 KB of flash memory. It also has an external 256 KB static random access memory (SRAM) chip, which is connected to the micro-controller using an external bus interface (EBI). Unlike Linux-based modular computers, the M-series micro-controllers are extremely power-efficient, with the ability to transfer data in low-energy modes using direct memory access (DMA). By using a circular buffer data structure together with DMA, partitions can fill from the microphone without CPU intervention, thus reducing energy requirements. The circular buffer also allows memory partitions to fill from the microphone while other partitions are writing to the microSD card or running a detection algorithm on collected samples. Therefore, there is no loss of data between writes or analysis, meaning the AudioMoth is able to record or listen continuously.

### 2.2. Firmware

The firmware used on AudioMoth can be updated or replaced by the user. This could be the default open-source firmware or the user’s own custom firmware based on the publicly-available source code. Updates and firmware changes can be performed without additional development boards, requiring just a USB cable. Users can adapt basic functionality to incorporate their own detection methods, or implement commonly used post-processing techniques to reduce storage consumption, such as storing species occurrences [20]. They can also attach components such as additional sensors to the PMOD header on the face of an AudioMoth and implement its functionality using custom firmware. While devices based on the Raspberry Pi, such as Solo [16], are simpler to configure in higher-level languages such as Python, AudioMoth sacrifices this simplicity in exchange for lower-level control of the hardware and is programmed in C with no operating system.

The device’s firmware was written to work in tandem with a custom-designed USB configuration application, built using the Electron framework [21]. The application is designed to make the process of configuring AudioMoth user-friendly for less technically skilled users. It enables users to set up a deployment specific configuration, setting the sample rate, setting the gain level of the on-board amplifier, and scheduling both recording and sleeping periods throughout the day. This makes it easy to set up AudioMoth for multiple uses with the default firmware, requiring minimal additional expertise or training. All firmware and supporting software of this type are open-source to encourage further development on the AudioMoth platform, with the code and hardware designs available under open licenses.

## 3. Detection Algorithm Applications

Acoustic detection is a growing area of bioacoustics and is used to both replace and supplement existing environmental monitoring methods, such as camera trapping and physical surveys [4]. Acoustic analysis is now an established approach to detect insect pests in grain and wood due to the ease of discerning vibrations in solid substrates [10,22]. However, the complexity of acoustic detection problems increases in air, with more competing sounds and more rapid attenuation of signals. This means that acoustic detection methods operating in air must be more complex in response.

Acoustic analysis algorithms utilised for tasks such as species identification or soundscape analysis will often make use of computationally intensive processes, such as complete fast Fourier transforms (FFT) or elaborate spectral analysis, such as calculating mel-frequency cepstrum coefficients (MFCC) [23]. These tasks are typically performed after all data has been collected, so powerful desktop machines can be utilised or they can be done over a long timescale, reducing the importance of their computational complexity.

An increasingly popular method of analysing large acoustic datasets is the use of deep learning techniques, such as convolutional and recurrent neural networks trained on large, previously collected sets of recordings. Recurrent neural networks lend themselves to audio analysis by assuming that features represented in the past will also affect future observations [24]. This is useful when detecting acoustic events that consist of distinct, successive sounds, such as complex insect or bird calls. These deep-learning techniques have been applied to useful effect in the classification of both insects [25] and birds. The majority of responses to the bird classification challenge issued by Stowell et al. [26] utilised some form of deep learning to complete the task [27,28,29].

Neural networks designed for animal classification often outperform commercial classification systems in terms of accuracy. One example of a system which does so is Bat Detective [20], a convolutional neural network designed to detect bat echolocation calls. It was trained on a large dataset of full-spectrum bat recordings collected by citizen scientists at road transects throughout Europe. The network was run on a desktop computer with an Intel i7 processor and 32 GB of RAM to make it possible to analyse sets of high-sample-rate recordings in real time. Unfortunately, it is not possible to produce an acoustic monitoring device capable of running a neural network such as this in real time without sacrificing both the low-cost and low-power requirement.

On-board detection on low-cost hardware with limited computing power, such as on AudioMoth, must still be done in real time, as the window for calculation is constrained to the time it takes to collect the samples. Despite this, performing any analysis on the device itself yields significant advantages. A detection algorithm that decides whether or not to record reduces the size of datasets collected over the course of a deployment and improves the energy efficiency of the recording device. On AudioMoth, detection algorithms are processed after a set of samples have been collected in the device’s memory but before they are saved to the microSD card. The act of saving a recording is energy intensive, compared to energy use while listening and analysing. Depending on the model of card used, AudioMoth uses up to 40 mA when writing to the microSD card and as little as 5 mA when listening and storing samples temporarily in memory. Real-time detection also reduces storage requirements, as less storage is wasted on unwanted recordings, which would have to be filtered out later. As well as this, retrieving data from sensors is time-consuming and difficult in isolated areas with rough terrain; therefore, requiring fewer trips to the deployment sites for data collection can save large amounts of time and manpower.

### 3.1. Frequency Component Extraction

**Figure 2:**
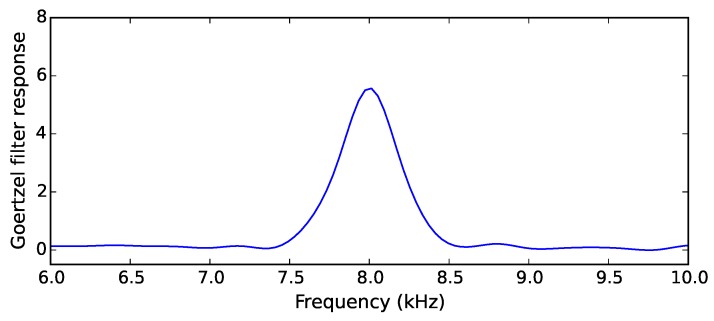
An 8 kHz tone extraction with a single Goertzel filter.

A step common to all acoustic detection algorithms is the extraction of features which define the target sound. The presence or absence of certain frequency components in a sound is one such feature. In order to perform frequency component extraction without the computational expense of frequently running complete fast Fourier transforms (FFT), the Goertzel algorithm is used to evaluate a subset of terms of a full FFT. A filter employing the Goertzel algorithm produces a response which represents the presence of a band of frequencies in a signal, shown in Figure 2. This frequency band can vary in width around a chosen central frequency depending on the target sound, as well as the application. We use different numbers of Goertzel filters of varying lengths and bandwidths in each of the three described algorithms, with subsequent filters and analysis techniques also differing between each algorithm.

To apply the Goertzel algorithm to a set of samples and obtain frequency terms for a central frequency *f* and a bandwidth *B*, the samples are divided into *N* windows of length *L*, such that they are denoted $\left(s_{1,1},\ldots ,s_{1,j},\ldots ,s_{1,L},\ldots ,s_{N,1},\ldots ,s_{N,j},\ldots ,s_{N,L}\right)$ where 1≤i≤N and 1≤j≤L. The length of a Goertzel filter *L* depends on the sample rate $f_{s}$ and the desired bandwidth *B*:

(1) $$L=4\frac{f_{s}}{B}$$

A temporary sequence of values *y* is obtained for a filter using a constant *c* and Hamming windowed data $h_{j}$:

(2) $$y_{i,j}=\left(h_{j}\cdot s_{i,j}\right)+\left(c\cdot y_{i,j-1}\right)-y_{i,j-2}.$$

In this equation, *c* is pre-computed using the target frequency *f* and the sample rate $f_{s}$, and is given by:

(3) $$c=2\cos\left(\frac{2\pi f}{f_{s}}\right)$$

While the filter is running on a finite signal, a Hamming window is applied in order to prevent spectral leakage. The values used by the Hamming window can be also be pre-computed, given a fixed filter length *L* and two constants α=0.54 and β=1−α=0.46:

(4) $$h_{j}=\alpha -\beta \cos\left(\frac{2\pi j}{L}\right)$$

Each filter generates a magnitude $m_{i}$ from the temporary sequence, expressing the presence of the target frequency band in those samples:

(5) $$m_{i}=y_{i,L}^{2}+y_{i,L-1}^{2}-c\cdot y_{i,L}\cdot y_{i,L-1}$$

This output is then passed to the detection stage in each of the three algorithms that we describe. Calculating the magnitude of a target frequency within a set of samples in this way is computationally less complex than a discrete Fourier transform (DFT). With *L* samples in a window, the computational complexity of performing the Goertzel algorithm on that window is O(L), whereas a DFT on the same sample count is calculated to be O(LlogL). The number of samples in a window is either 128 or 256 in each of the three algorithms described in this paper.

### 3.2. Bat Detection Algorithm

This algorithm is designed to detect various species of bat by listening for their echolocation calls. These calls vary in terms of structure and central frequency, but are generally short, ultrasonic and occur multiple times in quick succession. Once a call has been detected, a recording is produced for later identification and analysis.

Most species of bat forage at various times throughout the night, meaning daily sampling must last for at least 12 h. Due to the high energy and storage usage when recording at a sample rate high enough to capture ultrasonic frequencies, the algorithm’s performance has a significant effect on the device’s ability to remain active for a reasonable amount of time when deployed in remote locations. If each device recorded continuously, rather than using a detection algorithm, it would require 18 GB of storage each night and would exhaust the energy supply of three AAA-cell batteries in approximately three nights.

#### 3.2.1. Detection Algorithm Requirements

Different species of bat produce calls that vary within the band of 11 kHz to 200 kHz [30]. However, the majority of echolocation calls exist in the ultrasonic range of 22 kHz and higher. In order to capture the ultrasonic band in which most bat calls are found, AudioMoth was set to listen and record at a sample rate of 250 kHz. The targeted detection frequency of the algorithm can be set between 0 and 125 kHz given this sample rate, allowing for the detection of calls from a wide variety of bat species.

#### 3.2.2. Sample Collection

For this implementation, the external 256 KB SRAM buffer held a single partition containing 128,000 samples, representing 512 ms of audio. The algorithm ran on a duty cycle, waking every two seconds to run through a single partition of captured samples. This sleep period was included to improve the battery life of devices running the algorithm and collecting samples at such a high sample rate. Each iteration of sleeping and listening lasts approximately 2.5 s.

#### 3.2.3. Feature Extraction

One Goertzel filter, centred at a frequency chosen based on the target species, with a bandwidth of 4 kHz, was applied once the single partition had been filled. The chosen frequency band should be in the centre of the range of frequencies present in the target echolocation call in order to allow for variations in the upper and lower limits of a call. To achieve this bandwidth, the filter requires 256 samples (L=256). The 128,000 samples were divided into 500 windows (N=500), producing a set of 500 amplitudes.

#### 3.2.4. Detection Stage

**Figure 3:**
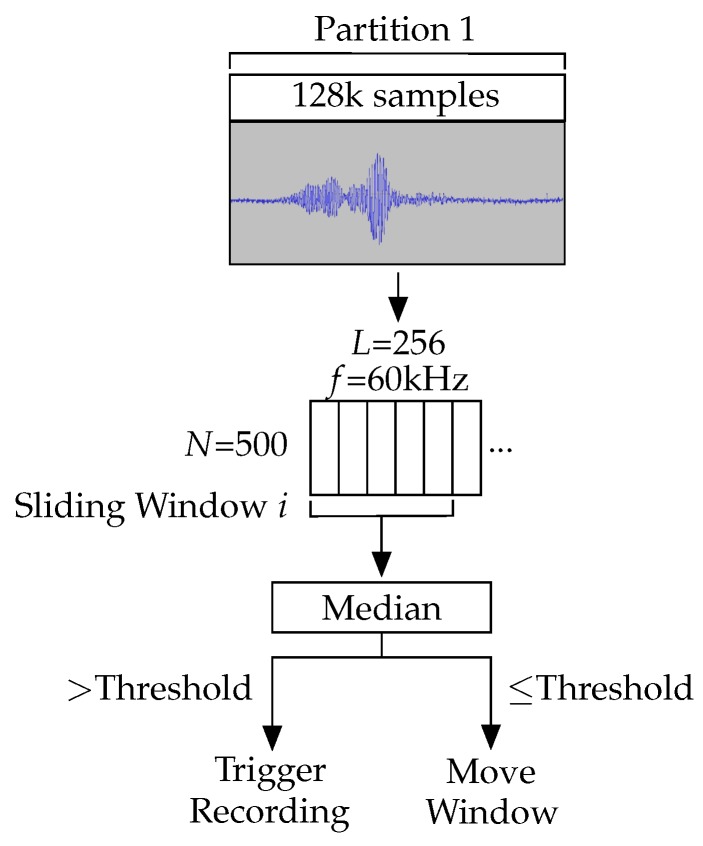
Overview of the data collection and bat detection process. Samples are collected and stored in a partition of the SRAM. Once full, the collected samples are fed into a 60 kHz Goertzel filter in windows of 256. A sliding window is run over the resulting series of responses. The sliding window takes the median response from five Goertzel filters and compares it to a threshold. It then either triggers a recording or moves the window along.

**Figure 4:**
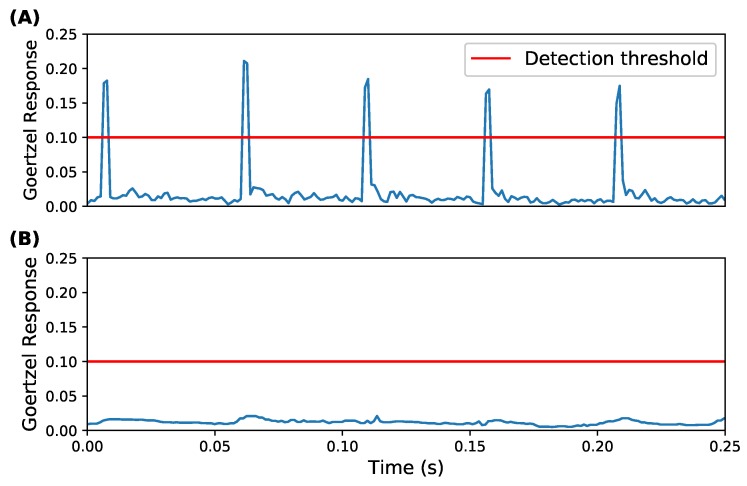
Bat detection analysis on a recording of a soprano pipistrelle bat, collected by a deployed AudioMoth device. The two plots each show median responses of two sliding windows, (**A**) with the correctly configured five Goertzel outputs per window; and (**B**) with too many, at 15 per window. Bat calls are missed when over half the windows consist of background noise, rather than the call itself.

Each echolocation call for the target species lasts approximately 5 ms. At a sample rate of 250 kHz, a 5 ms call consists of 1250 samples, which the detection algorithm is tasked to react to. A partition containing a single call will also contain a large of number samples of background noise. Thus, the algorithm employs a sliding window to minimise the likelihood of a call being lost in noise. The sliding window moves across the Goertzel filter outputs with a step size of half its width, processing each output two or more times. We obtained the median response of the values covered by the sliding window. A threshold was applied to each median, deciding whether to record or step the window forward. The threshold was chosen based on the response from recordings of the target species when they produce echolocation calls. The full detection process is shown in Figure 3. Appropriately configuring the sliding window produces pronounced responses to bat calls, despite the low signal-to-noise ratio in the captured samples. Note that the length of the sliding window must be tuned to the length of a single bat call. It cannot exceed twice the length of a call, since the call would then be split across two windows and it would fail to produce a median response to trigger the threshold. This is shown in Figure 4 where the median smooths out the target frequency responses. Conversely, if the window length is too short, redundant calculations were performed in addition to the resulting identifier being noisier.

As Goertzel filter outputs can be calculated once and stored for use in multiple windows, it is not necessary to apply the filter to the entire window every iteration. Outputs can be briefly stored until their use in a subsequent operation. The majority of the additional computation when analysing every window of samples multiple times comes from combining the values into a single median.

#### 3.2.5. Performance

The accuracy of the detection algorithm was assessed using a set of recordings of the soprano pipistrelle (*Pipistrellus pygmaeus*), a UK bat species which produces echolocation calls with a strong 60 kHz frequency component. This dataset consists of 238 recordings, split between the soprano pipistrelle and background noise.

The number of true positive, false positive, and false negative responses to each set of recordings was used to calculate the precision and recall of the algorithm. Precision is a value representing the fraction of correct positive responses among all responses. Recall is a value representing the fraction of correct positive responses among all possible positive responses.

(6) $$\mathrm{precision}=\frac{\mathrm{true}\;\mathrm{positives}}{\mathrm{true}\;\mathrm{positives}+\mathrm{false}\;\mathrm{positives}}$$

(7) $$\mathrm{recall}=\frac{\mathrm{true}\;\mathrm{positives}}{\mathrm{true}\;\mathrm{positives}+\mathrm{false}\;\mathrm{negatives}}$$

Using both precision and recall, the *F*${}_{1}$ score was calculated as follows:

(8) $$F_{1}=2\times \frac{\mathrm{precision}\times \mathrm{recall}}{\mathrm{precision}+\mathrm{recall}}$$

This metric was used in the assessment of each of the three algorithms described, and a set of target sound recordings and background noise recordings were collected for each. The bat detection algorithm obtained an *F*${}_{1}$ score of 0.961 from a precision of 0.994 and a recall of 0.931. This high level of accuracy is due to the absence of possible false positive sources in the ultrasonic frequency range being targeted. The overall background noise in the UK dataset obviously differs from the background noise present in other possible deployment locations; however, likely sources of false positives in the targeted ultrasonic band will be similar (frogs, other bat species).

**Figure 5:**
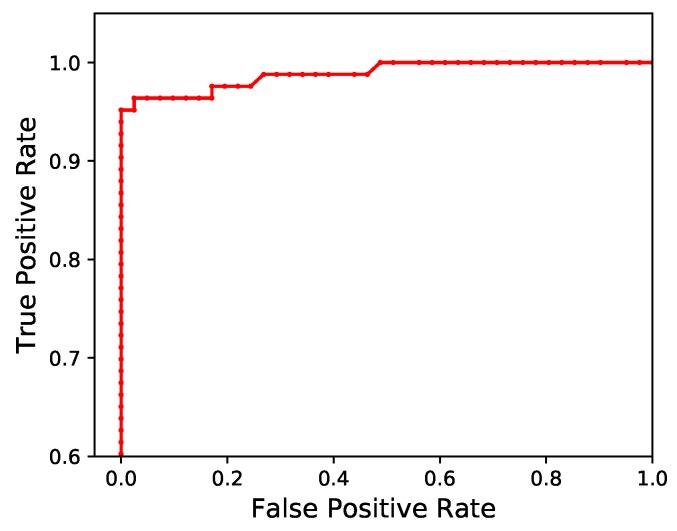
A receiver-operating characteristic curve showing the true-positive and false-positive rate produced by a range of thresholds when used by the bat detection algorithm.

Figure 5 shows the receiver operating characteristic (ROC) curve over a range of thresholds. Using the dataset used to calculate the *F*${}_{1}$ score, the bat detection algorithm produced an area under the ROC curve (AUC) of 0.975 (very close to the optimal value of 1).

When deployed, the algorithm-equipped devices cycled through sleeping for two seconds, collecting samples for 512 ms and running the analysis for 276 ms. In terms of energy consumption, AudioMoth used 45 μA while sleeping, 20.94 mA when collecting samples, and 17.65 mA during analysis.

#### 3.2.6. Deployment

A deployment of this algorithm was carried out by the Zoological Society of London (ZSL) as part of a preliminary acoustic survey of a species of bat endemic to Cuba. They required sensors to make 10 s recordings in response to the call of the Cuban greater funnel-eared bat (*Natalus primus*). Using the recordings, they aimed to reveal the species’ foraging distribution, as well as their habitat preferences.

**Figure 6:**
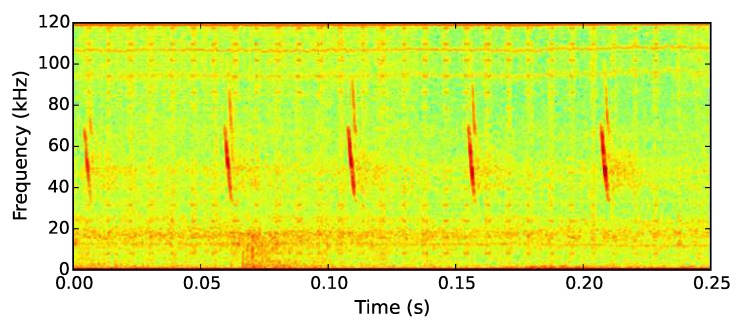
Spectrogram showing the first target sound: echolocation calls of a soprano pipistrelle bat.

The echolocation call of the Cuban greater funnel-eared bat contains a pronounced 60 kHz frequency component, similar to that of the soprano pipistrelle bat (see Figure 6). Recording at 250 kHz captured not just this prominent frequency component but the majority of the ultrasonic call. The bat detection algorithm was deployed on 20 AudioMoth devices in the Guanahacabibes peninsula. The trial deployment covered the area surrounding the single known roosting cave used by the species (Cueva la Barca) [31]. The devices were deployed along four transects radiating from a single exit at 20, 40, 60, 180, and 360 m for a total of three nights in order to investigate the foraging distribution of the bats, as well as their habitat preferences.

The devices were set to record between 5:30 p.m. and 7:00 a.m. (45 min before sunset and 10 min before sunrise), triggering and recording a dataset of bat calls and the distance from the cave each occurred. Each device was equipped with AAA-cell (1200 mAh) batteries, allowing them to listen for approximately 16 days if set to listen for 13.5 h, remaining in a low-power sleep mode for the remaining 10.5 h each day.

The data was collected from 18 deployed devices (two device failures occurred due to user error and single hardware failure) and using the detection algorithm, the Zoological Society of London successfully captured 25 h of recordings featuring the Cuban greater funnel-eared bat. Had the devices simply recorded during the listening intervals, then over 729 h of data would have been collected, incurring significant additional manual filtering time and expense. The ZSL research team plan to use the deployed devices to track the movement of groups of foraging bats throughout the surrounding forest, extending the survey area outwards in future, and extending the survey duration to make best use of the AudioMoth devices’ capabilities.

### 3.3. New Forest Cicada Detection Algorithm

This algorithm was developed to enable AudioMoth to automate the search for the possibly locally extinct New Forest cicada (*Cicadetta montana*) [32]. The cicada is considered a priority species by the Joint Nature Conservation Committee since 2017, being the UK’s only native cicada [33]. As its last confirmed sighting was over 22 years ago, its conservation status in the UK is currently unknown [34]. Outside of the UK, the species is found throughout Asia and continental Europe, and in various countries, including Slovenia [35].

#### 3.3.1. Detection Algorithm Requirements

Male New Forest cicada sing with an extended buzz lasting around 30 s with a dominant frequency of 14 kHz [36]. This is a frequency rarely present in the calls of other species found in the New Forest. One such exception is the dark bush cricket, which calls at the same frequency as the New Forest cicada. Despite the shared frequency component, their calls are easily distinguished as the cricket’s consists of a series of intermittent, broad-spectrum chirps. Wind also generates significant noise at 14 kHz. Distinguishing between a cicada call and wind noise is done through use of a second frequency component at 8 kHz. If a cicada is detected, we require a prolonged recording of the call for post-deployment analysis to verify the trigger as a New Forest cicada. Thus, a 30 s recording is made upon triggering of the algorithm.

#### 3.3.2. Sample Collection

Listening for cicadas requires continuous monitoring from late spring to early summer, to cover the emergence of adults. The feasibility of the three-month deployment therefore depended crucially on the performance of the algorithm in maximising battery life. Between listening/recording periods, the algorithm puts the device to sleep to conserve battery. For this application, AudioMoth’s SRAM is configured to use a single partition of 8192 samples at 48 kHz. This constitutes listening for 171 ms. The 5 s sleep period, combined with the listening period creates a short duty cycle which can repeat multiple times over the course of a 30 s cicada call. This enables multiple opportunities for detection. Using this design to detect longer vocalisations increases likelihood of detection without adding extra complexity to the detection process itself.

#### 3.3.3. Feature Extraction

Once the partition has been filled, the samples are fed into two Goertzel filters. Each of these filters uses 128 samples (L=128) to achieve a bandwidth of 1.5 kHz around the two different central frequencies. The 8192 samples are fed through the filters as 64 windows (N=64). One set of filters extracts the 14 kHz component to detect the cicada call itself, whereas the other extracts the 8 kHz component to filter false positives caused by wind noise.

#### 3.3.4. Detection Stage

**Figure 7:**
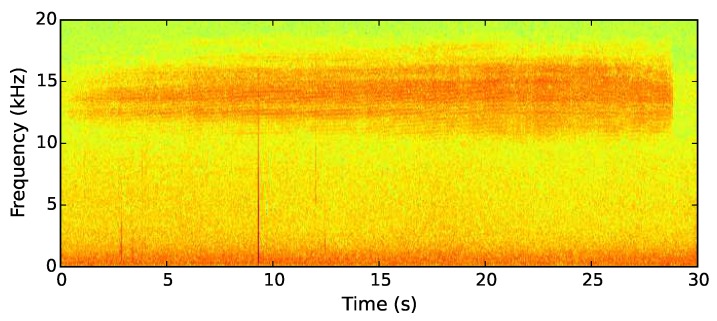
Spectrogram showing the second target sound—the song of the cicadetta montana.

The output values from each of the two sets of filters are zipped together to create 64 pairs of Goertzel responses. The ratio of each pair is then calculated. The ratio between the 14 kHz frequency component and the 8 kHz component of each sample produces an identifier robust to broad spectrum noise. Wind produces noise at both 14 and 8 kHz, so by using a ratio as the identifying value, wind noise can be distinguished from cicada calls through their differing responses. A recording is only triggered when a high 14 kHz value and a low 8 kHz value produce a high ratio. This is more likely to occur when a cicada produces a call as it will cover 14 kHz, but not 8 kHz (see Figure 7). The group of samples is more likely to contain wind if a high value is calculated for both frequencies, generating a low ratio.

**Figure 8:**
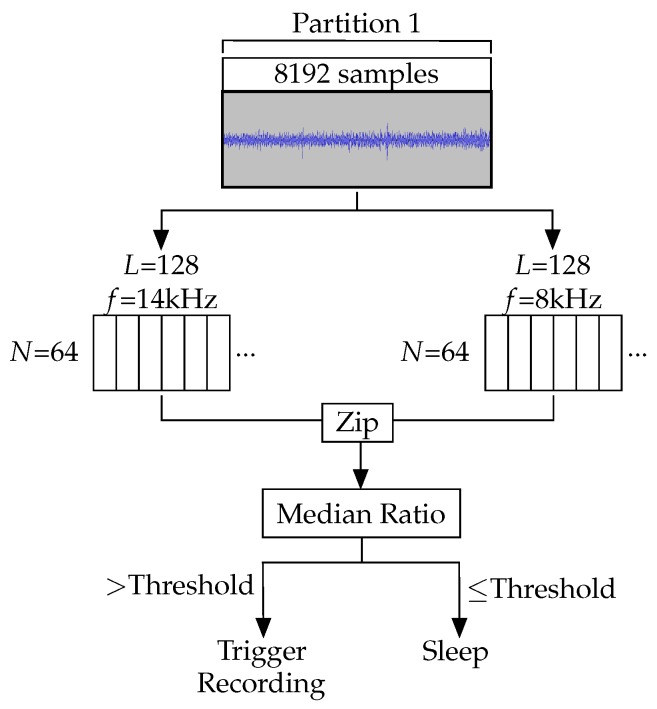
Overview of the sample collection and cicada detection process. Samples are collected in a single partition of the SRAM containing a maximum of 8192 samples. Once it is full, the samples are fed through two Goertzel filters in windows of 128 samples. The outputs from these filters are zipped together and the ratio of each pair is calculated. The median value of these ratios is compared to a threshold to decide either to record or return to sleep.

The median value for the set of 64 ratios is compared to a threshold. If a cicada call is detected in a partition, a 30 s recording is made. If it is not detected, the device returns to sleep. This process is shown in Figure 8.

**Figure 9:**
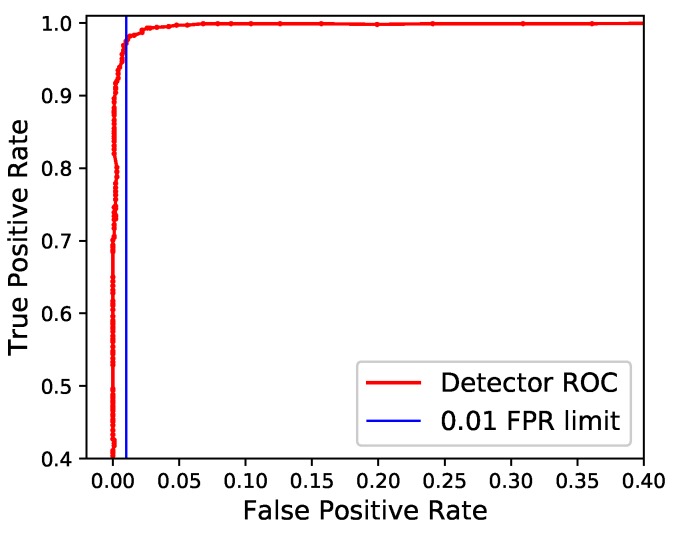
Receiver operating characteristic curve showing the performance of the New Forest cicada detector at various thresholds. The final threshold was chosen by capping the false-positive rate to 0.01 and taking the threshold which corresponded with the highest attainable true-positive rate.

To set the detection threshold, a ROC curve was created (Figure 9) using a set of recordings of the cicada, gathered in Slovenia. As each point represents the true-positive and false-positive rate associated with a given threshold, selecting a point on the curve allows for a level of accuracy to be targeted. Because AudioMoth listens multiple times over the course of a 30 s call, a cicada has a high chance of detection even with a low true-positive rate, while a high false-positive rate will unduly limit the operational life of the device. By using a threshold with a false-positive rate of 0.01, approximately 60 false recordings can be expected when listening for eight hours per day.

For the deployment, each AudioMoth was deployed with a Kingston Class 10 microSD card, chosen due to its cost and write speed. The write speeds of microSD cards vary significantly and can have a major effect on overall energy consumption when a large quantity of recordings is expected. Recording to this microSD card consumes approximately 24 mA. With this level of energy consumption, an AudioMoth using a threshold corresponding with a false positive rate of 0.01 is calculated to operate for approximately 179 days with standard AA-cell (3000 mAh) batteries. With a false-positive rate of 0.05, this drops to 48 days. In order to ensure sufficient battery life for a deployment lasting three months, the false-positive rate was capped at 0.01. Making the device less proactive in terms of data collection in this way will result in a higher false negative rate, however this trade-off was considered when designing the deployment. By deploying the devices with overlapping detection areas, it was possible to further compensate for possible false negatives by having multiple devices covering each section of the chosen sites, resulting in multiple chances to detect any calls.

#### 3.3.5. Performance

As with the bat detection algorithm, the precision, recall, and *F*${}_{1}$ score metrics were used to assess the accuracy of the New Forest cicada detection algorithm. These metrics, as well as the battery life compared to the requirements of the deployment, were used to assess the overall effectiveness of the algorithm for this application. To obtain the *F*${}_{1}$ score, a dataset of 196 cicada recordings, as well as 228 recordings of background noise was collected from a known Cicadetta montana site in Slovenia. Using the equations described in (6), (7), and (8), the cicada detection algorithm achieved an *F*${}_{1}$ score of 0.982 from a precision of 1.0 and a recall of 0.964. The ROC curve used to select the threshold was also used to calculate the area under the curve as an accuracy metric. For the cicada detector, this was 0.998. This high level of accuracy is a result of the detector being insensitive to many possible sources of false positives within the dataset, such as wind noise or other insects.

The high level of accuracy attained by our algorithm, combined with a predicted lifespan from 3 AA-cell batteries (3000 mAh) exceeding the three-month requirement of the application allowed us to judge the algorithm as suitable for long-term deployment.

#### 3.3.6. Deployment

The algorithm to detect the New Forest cicada was first deployed on 87 AudioMoth devices, sited in four locations for a three-month period during late spring to early summer in 2016 in the New Forest National Park, UK. To maximise the chance of detection, the devices were positioned in locations chosen by entomologists who previously conducted annual surveys of the area with direct sampling methods. These sites were selected throughout the New Forest and were chosen to be off walking routes commonly used by the public. Historically, this made manually surveying these locations throughout the cicada’s active period a challenge. The deployment process was repeated with a reduced number of devices covering the same sites in 2017 and 2018. After each deployment, the recordings collected by the devices were manually inspected for evidence of the cicada.

The search for the New Forest cicada is an ongoing case study that makes use of AudioMoth’s ability to monitor a large area of woodland at a low cost. None of the three AudioMoth deployments have yet resulted in evidence of the New Forest cicada in the area. However, the combination of AudioMoth and the cicada detection algorithm has improved the spatial and temporal coverage of monitoring efforts. Large areas of the forest have been constantly monitored for the entire deployment period for three years, requiring just two annual trips to each site for deployment and collection, rather than relying on manually surveying areas at regular intervals.

### 3.4. Gunshot Detection Algorithm

Conservation biologists need to monitor rates of natural resource extraction in order to evaluate the vulnerability of natural areas to biodiversity loss. This algorithm was designed to detect gunshots as part of an effort to monitor poaching in a tropical forest reserve in Belize, Central America.

To characterise gunshots and to identify features to inform the basis of detection, we studied sound characteristics of gunshots from two different types of shotgun (12 and 16 gauge) fired at various distances along a transect in mature forest at Pook’s Hill Reserve. This set of ground-truthing data allowed us to identify the characteristics of gunshot sound propagation that form the probability distributions used by the model.

#### 3.4.1. Detection Algorithm Requirements

**Figure 10:**
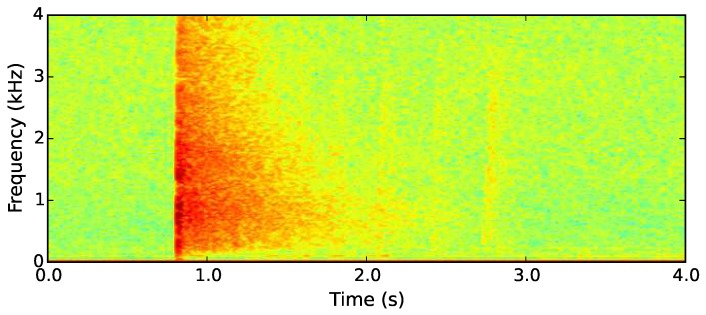
Spectrogram showing the third target sound—a gunshot recorded in the rainforest at a distance of 255 m.

For all types of shotgun, the initial muzzle blast is a loud impulse which covers a wide range of frequencies when recorded close to the source [37]. As the gunshot sounds propagate from their source, their high-frequency components decay as they are absorbed into the air and the surrounding dense rainforest (see Figure 10). A gunshot can be detected by characterising the rate at which selected frequencies peak in the initial muzzle blast and subsequently decay.

While the bat and cicada detection algorithms rely solely on the frequency domain, gunshots require analysis covering the time domain in order to utilise the signature transition from muzzle blast through different stages of decay. By using the time domain, the algorithm is able to distinguish a gunshot from an insect coincidentally vocalising in the same frequency band. For this reason, the algorithm uses a HMM with three frequency components as observations.

#### 3.4.2. Sample Collection

Poaching of game animals in the target area in Belize occurs almost exclusively at night. This means that monitors need to sense the environment for around 12 in every 24 h, between dusk and dawn. Unlike both the cicada and bat detection algorithms, the gunshot detection algorithm has no expectation that the target sound will immediately repeat after it has been detected once. For this reason, no duty cycle routine is used and the deployed devices listen constantly during night-time hours despite the energy cost of doing so. In order to compensate for this and extend the battery life of the device, the processor clock was reduced from AudioMoth’s default of 48 MHz to 11 MHz.

As with bat echolocation calls, the target sound of the gunshot detector is short, with a gunshot lasting approximately one second before decaying to the point of being inaudible. For this reason, samples are collected constantly, using a three-partition circular buffer with each iteration of the detection algorithm running on two of the three partitions. The detection algorithm is applied to the first and second partitions in the time taken to fill the third. This means that all partitions are included in two iterations of analysis. Therefore, the full gunshot decay structure the algorithm attempts to identify will be detectable even when the samples containing it are split across partitions.

Each partition contains 16,384 samples collected at a sample rate of 8 kHz. As with cicada detection and unlike bat detection, it is not necessary to record at a sample rate capable of covering ultrasonic frequencies. As each iteration of the detection algorithm uses two partitions, analysis is done on 32,768 samples which represent 4.096 s of audio. As the gunshot is not likely to be immediately repeated, the samples used for analysis are stored, rather than a new recording being made following the detection algorithm response.

#### 3.4.3. Feature Extraction

The model uses three frequency components as features, and they were extracted by Goertzel filters at 400 Hz, 1200 Hz, and 2000 Hz. Recording at a sample rate of 8 kHz, we used Goertzel filters of length 128 (L=128) to achieve the desired bandwidth of 250 Hz and pass through the 32,768 samples as 256 windows (N=256). The chosen frequency components represent the decay pattern of the sound without relying on higher frequencies which will have decayed by the time the sound has reached the sensor.

#### 3.4.4. Detection Stage

**Figure 11:**
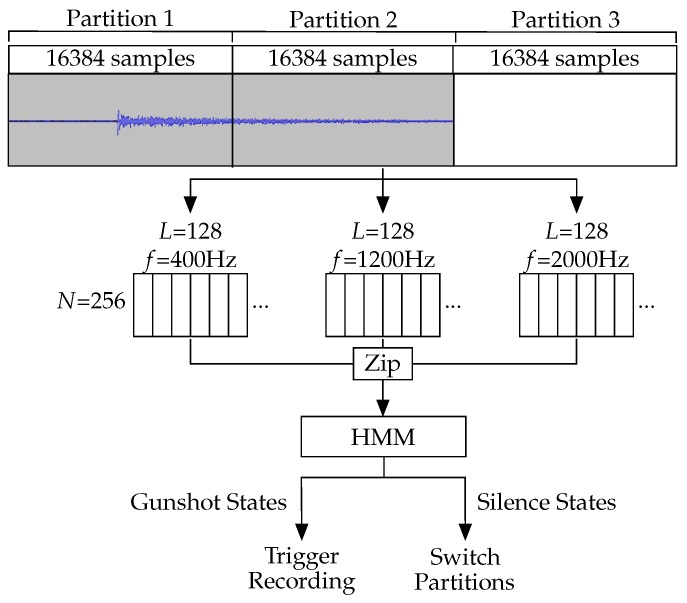
Overview of the data collection and gunshot detection process. Samples are collected and stored in three partitions in SRAM. Once two have been filled, the detection algorithm is run while the third fills. Once the third has been filled, the algorithm is run again on the most recent two partitions. If a gunshot does not fit into a single partition, each partition is used twice meaning the full gunshot will be in the next iteration.

Once all three Goertzel filter outputs have been generated, they are zipped together into 256 sets of three values. These triples are fed into the HMM and treated as state observations to produce a series of 256 states. This process is shown in Figure 11.

**Figure 12:**
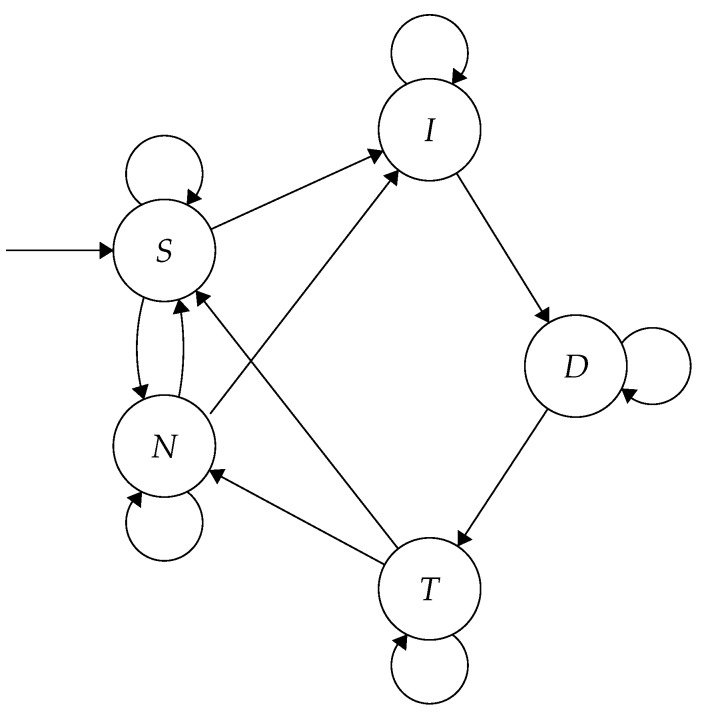
Gunshot detection model state diagram showing possible movement between five states: silence *(S)*, noise *(N)*, impulse *(I)*, decay *(D)*, and tail *(T)*.

The HMM classifies the Goertzel outputs into five possible states: Initial impulse *(I)*, decaying impulse *(D)*, tail *(T)*, noise *(N)*, and silence *(S)*. Movement between these states is shown in Figure 12. As the Goertzel-filtered samples are run through the HMM, the Viterbi algorithm [38] is used to predict the most likely path through the states taken by the recording. A gunshot is detected if the predicted states run through all three gunshot states (*I*, *D* and *T*). When this occurs, the samples in the two current SRAM partitions are stored on the microSD card as an uncompressed WAV file. This contrasts with the previously described algorithms which start recording following a detection. The buffers that triggered the algorithm contain the possible gunshot, whereas following samples will only contain evidence of one in the unlikely event another gunshot occurs in the four seconds immediately proceeding it.

We used log-normal distributions for the emission probabilities of the HMM. These distributions possess long tails which can account for extreme responses with high magnitude, such as when a recording is made close to the source of the gunshot. The mean and variance for each of the five states is calculated from labelled samples within a set of gunshot recordings. If the devices have sufficient coverage of an area and a shot is fired, it is likely that one device will be close to the source and produce a result represented in the extreme of the distribution. In order to prevent extreme values from exclusively favouring a specific state, all probabilities were normalised such that the probability of any single state is no more than 100 times more than that of any other.

To calculate the transition probabilities we took a representative set of gunshot recordings, manually labeled the points within the shot where they transition between states, and used the mean time spent in each state of the gunshot. These probabilities were tested by simulating gunshots, randomly producing sequences of states according to the transition probabilities, and ensuring that the sequences had a similar form to those seen in real gunshots.

#### 3.4.5. Performance

To quantitatively assess the accuracy of the gunshot detection algorithm as we have with the previous algorithms, we used a set of recordings collected in 2017 containing gunshots fired between 0 and 800 m during both day and night in the Belizean rainforest. Constant recordings within the test area were divided into 4 s sections and manually classified as containing a gunshot or just background noise, producing a dataset containing 1,170 positive recordings. The algorithm was run on the dataset, resulting in an *F*${}_{1}$ score of 0.75 from a precision of 0.92 and a recall of 0.64. The lower recall is due to the higher number of false negatives on gunshots far from the recording device. With longer distances for the sound to travel, the distinguishing features of the gunshot were lost as higher-frequency components were absorbed by the environment or drowned out by background noise.

The background dataset contained possible sources of false positives in the form of branches snapping, insect calls, and bird calls. On 2970 background recordings, the detector produced 240 false-positive classifications (an FPR of 0.08), triggering on uncommon events such as insect calls immediately adjacent to the device or loud human speech, which could not be represented within the noise state emission probabilities.

While running the AudioMoth clock at 11 MHz, the algorithm took 1070 ms to complete, using just 6.48 mA while doing so. This used up slightly more than half the available time while the next two seconds of audio were collected. If deployed using AA-cell batteries (3500 mAh) as before, the predicted lifespan was just 53 days. However, when deployed using a 6 volt battery (26,000 mAh) the predicted lifespan increased to 398 days.

With an FPR of 0.08 the detector produces approximately 145 false positive recordings per hour, requiring 109 MB of storage each night. While such a high volume of false positives would not work for a system designed for live gunshot reporting, it is sufficient for logging deployment and is a significant improvement over recording continuously every night. A continuously recording device would produce more than six times the quantity of recordings per hour and have a predicted lifespan of 214 days with a 6 volt battery. In terms of storage, this device would require 659 MB of storage for each night.

In terms of computational complexity, a HMM is calculated to be $O(S^{2}N)$, in which *S* is the number of states in the model and *N* is the length of the observation sequence. In the case of this model, there are five states and the observation sequence length is the number of Goertzel filter outputs from the feature extraction stage (N=256).

#### 3.4.6. Deployment

We tested the first iteration of our gunshot detection algorithm in Pook’s Hill Reserve. This iteration did not possess a dedicated noise state—instead, it classified all non-gunshot samples as silence. The deployment included 36 devices along a transect, with each deployment point possessing two devices facing in opposite directions. Each location was positioned 200 m apart and a series of 65 controlled gunshots were fired in both directions along the line of the transect using a 12-gauge (Baikal MP-18EM-M) and 16-gauge (Rossi single shot) shotgun. By firing the gunshots in both directions at varying locations we collected both directional and distance data. This study aimed to identify how these variables affect the gunshot detection ability of AudioMoth and the first iteration of its detection algorithm in tropical forests.

On gunshots fired at ranges up to 800 m through dense, broadleaf rainforest, the detection algorithm responded to and recorded gunshots with a true positive rate of approximately 0.57. With the recordings collected during this deployment, the model was reconfigured, using the samples to form a new set of emission and transition probabilities as well as produce the test dataset to benchmark future developments of the algorithm.

Subsequent iterations of the algorithm also introduced the noise state to the HMM to accurately classify more instances of false positives, rather than attempt to encompass them within the silence state’s probability distributions. An iteration with a noise state and updated model distributions based on the new recordings was developed and tested in early 2018, before being deployed in the final monitoring locations in the Tapir Mountain Nature Reserve, adjacent to the Pook’s Hill Reserve.

This iteration of the model was tested in a wider array of terrain, measuring the detection accuracy with hills and valleys between the source gunshot and the test devices. For gunshots fired within 500 m of the AudioMoth, the algorithm achieved a true-positive rate of 0.84. Beyond this distance, factors such as terrain and foliage around the test location caused the accuracy to vary significantly (Table 1). To encapsulate the effects of these variables we measured the amplitude of the gunshot at the site of the AudioMoth. From this we found that when the amplitude of a gunshot heard at the sensor fell below 60 dB, the algorithm was unable to distinguish it from background noise. Background noise throughout the reserve varied between 33 and 55 dB when measured at the AudioMoth deployment location for each test.

### 3.5. Gunshot Detection Neural Network Comparison

Deep-learning techniques have become the gold standard within the field of acoustic analysis due to their flexibility and the availability of previously collected acoustic datasets. These techniques include deep [39], recurrent [40], and convolutional neural networks [41].

Convolutional neural networks (CNNs) are increasingly used in classification applications involving acoustics [20,27,42] in response to success in computer vision applications [43]. CNNs are well-suited to the task of acoustic detection, as variation in terms of amplitude across frequencies can be represented as spatial features in a processed spectrogram. These features are easy for humans to visually identify; thus, a detection method mimicking image detection in the human brain is a logical solution to this task, utilising 2D structures in frequency and time [44]. With a dataset containing a wide variety of gunshot examples and false positives, a CNN can be trained to identify features which define a gunshot and use these features to classify new sample sets.

The gunshot/background noise dataset created to train the HMM classifier was also used to train and test three convolutional neural networks, each of which used differently pre-processed audio as input data. These pre-processing techniques represented three commonly-used methods of preparing acoustic data for neural networks. The aim of these classifiers was to provide a performance comparison for the HMM classifier using one of the most commonly used deep-learning techniques applied to acoustic detection.

#### 3.5.1. Network Design

Samples were pre-processed by generating either standard spectrograms, mel-scale frequency bank images, or mel-frequency cepstrum coefficients (MFCCs). The mel-scale is commonly used in audio processing specifically for detection of sounds within the range of human hearing. It mimics the non-linear perception of sound experienced by the human ear, being more discriminative at lower frequencies and decreasing as frequencies increase [45].

Frequency banks and MFCCs were produced using the same initial steps, with MFCCs requiring the addition of a discrete cosine transformation (DCT) as a final step. To perform both these processing methods, first the audio samples were divided up to produce a series of overlapping frames. A fast Fourier transform was then applied to each frame, followed by a series of triangular filters, spaced non-linearly along the mel-scale. Frequency banks were obtained from these mel-scaled windows, whereas MFCCs required the application of a DCT, which broke the signal down into the sum of a series of cosine functions of various frequencies.

MFCCs previously gained popularity as the discrete cosine transformation decorrelates the filter bank coefficients [46]. This was a necessary step for machine-learning techniques based around techniques such as HMMs, which are highly susceptible to this correlation. Deep-learning techniques, such as convolutional neural networks, are less susceptible to correlated inputs, so the extra step is no longer as necessary; however, it is still widely used in speech detection applications [47].

Model size is an important factor to consider when deploying a neural network on hardware with limited storage capacity, such as AudioMoth (256 KB of flash memory). To reduce the size and complexity of the CNN, depthwise separable convolution was used in place of standard 3D convolution. This is a common method of creating compact CNNs for image classification [48,49] and involves convolving each RGB colour channel of the image separately, then using a pointwise convolution to combine them again. Doing so is more efficient than standard 3D convolution in terms of the number of operations and the number of parameters, thus reducing both the computational complexity and the overall size of the network [50].

#### 3.5.2. Training

The recordings collected to produce the HMM’s probability distributions represented gunshots at varying distances and in varying terrains, as well as a wide array of background noises which could produce false-positive results. The size of the datasets was increased by creating duplicate recordings but offsetting them in time by various amounts around the gunshot. This produced a larger dataset which represented cases where gunshots could occur at any point in the 4 s listening period. The dataset was then divided into a training set and a test set, selecting an equal number of recordings at each distance randomly.

#### 3.5.3. Performance

The CNN based around mel-scale frequency banks outperformed the networks based on alternative pre-processing techniques as well as the HMM in terms of both predicted false positives per hour and *F*${}_{1}$ score (Table 2). It achieved the highest *F*${}_{1}$ score due to its resistance to false-positive responses, shown by the high level of precision (0.97). While the mel-scale frequency bank CNN outperformed the HMM detector in terms of detection accuracy, it does so at the cost of both size and speed.

Even using depthwise separable convolution for each convolution layer of the CNN resulted in a 61.3 MB network. The AudioMoth’s Arm Cortex-M4F possesses just 256 KB of flash, insufficient for the depthwise separable CNN. Comparatively, the complete firmware to implement the HMM which achieved an *F*${}_{1}$ score of 0.75 is just 61.15 KB.

In terms of speed, the HMM was able to run on the same 4 s of audio approximately 300 times faster than the CNN due to its lower complexity. Producing the frequency bank images taken as input for the CNN uses a Fourier transform which has a computational complexity of O(LlogL). After pre-processing, the CNN applies 2D convolution on three layers of the network, and this convolution step has a computational complexity of O(WHkk), where *W* and *H* are the width and height of the pre-processed images (390 × 40 pixels for frequency bank images) and *k* is the size of the square kernel used to apply the convolution (the kernel size changes at each layer of the network). For both steps, the computational complexity of the CNN exceeds that of the HMM. As a result, CNN classification on an i7 desktop computer took on average 0.52 s to pre-process and classify a 4 s recording. In the same time period, 300 recordings could be classified using the HMM.

Scaling down the processing power of the hardware to that of AudioMoth, the HMM can be run in real time, classifying 4 s of audio in 1070 ms. A CNN which takes 300 times as long to complete would not run in real time. This means that listening would have to regularly pause, allowing the detection algorithm to catch up, while possibly missing gunshots.

Real-time acoustic keyword detection based on a neural network has been implemented on powerful Arm Cortex-M7 micro-controllers [51] which have a clock speed of 216 MHz. As well as increased clock speed, their energy consumption is approximately ten times that of AudioMoth’s M4 processor. This higher specification micro-controller also costs twice as much as the M4, as well as incurring extra expenses from the larger batteries required to achieve a lifespan sufficient for long-term deployments. This pushes a device capable of running a neural network for this application outside the realm of low-cost and low-power, given current hardware.

## 4. Discussion

The three algorithms that we have described here and their associated applications demonstrate how the use of efficient on-board detection can make large-scale and long-term monitoring feasible and financially viable for user communities with limited budgets. The combination of low-power detection algorithms and open-source hardware has been shown to be an effective solution to a number of real-world conservation issues. By recording in response to targeted acoustic events, monitoring tools are less constrained by energy and storage demands. Reduced requirements bring down the unit cost and size of acoustic sensors because smaller and cheaper batteries can be used in long-term deployments. By making both the software and hardware open-source, monitoring tools are more flexible, allowing them to be tailored to the requirements of specific applications. This can provide benefits in terms of affordability and storage/energy efficiency.

We show that versatile, open-source hardware such as AudioMoth can be tailored to a specific research project, without the high development costs involved in creating custom-designed hardware and software. Customising AudioMoth to a specific application can provide features and performance at significantly reduced costs when compared to commercial alternatives. This includes producing final datasets which require significantly less time for analysis. An AudioMoth adapted to act as a detector for a specific species of bird will only react to acoustic events that possess similar characteristics to the target species’ call, whereas a device recording constantly throughout periods where the bird is likely to sing will record any songs, plus hours of irrelevant audio.

In terms of future extensions, bringing the advancements in acoustic deep learning onto low-power hardware would drastically improve the flexibility of accessible acoustic detection. Given the availability of existing biophony and anthrophony datasets, detectors based on deep learning techniques could be trained for many ecological applications. A wide variety of deep-learning techniques for acoustic detection already exist, including various alternate network implementations (recurrent neural networks, deep neural networks, etc.) and pre-processing techniques. Development of deep-learning for this purpose has found use in applications such as detecting a wide variety of anthropogenic events from acoustic observations [52] and keyword detection on smart devices with constrained power budgets [51]. An extension to this work would be to explore these techniques, using the existing gunshot recording dataset to produce a model small and fast enough to run on low-power hardware, such as AudioMoth.

Despite the benefit of reduced development costs, any additional detection algorithms require coding ability in C, which is often beyond the immediate reach of environmental researchers wishing to use this technology. Users require a greater degree of programming ability if they are to develop and implement new detection algorithms of the sort described here. However, we aim to bridge this gap between user and technology, and enable less technically-skilled users to develop their own on-board detection algorithms, with solutions such as compilers that generate C code from higher level implementations of digital signal processing techniques. The consistent structure of the algorithms described in this paper show that steps such as sample collection and Goertzel filtering could be standardised to build a compiler for this task. By removing barriers involved in customising monitoring technology, we are aiming for a future where conservation researchers are able to easily tailor such devices to their applications. This customisation allows them to benefit from reduced costs and gain the ability to ask larger, more complex research questions.

Finally, we hope to expand the already extensive list of conservation projects AudioMoth has assisted in by iterating upon the design of the underlying AudioMoth hardware. We aim to do this in terms of improvements which provide greater computing power and more available memory while remaining both low-cost and low-energy. This can be achieved by using both a redesigned PCB layout and newer components with greater energy efficiency. The additional resources from these improvements allow for more elaborate computation in detection algorithms, such as implementations which incorporate more complex analysis techniques or perform detection on multiple target sounds simultaneously.

## Figures and Tables

**Table 1:** Detection counts and true-positive rates from shots fired at increasing distances from the AudioMoth deployment location. These sites varied in terms a wide variety of variables including terrain, wind direction, and foliage thickness, resulting in an array of detection accuracies at distances greater than 500 m.

Distance from Source (m)	100	200	300	400	500	600	700	800	900	1000
Trial shots	12	22	34	34	49	18	31	21	13	18
Detected shots	12	22	33	29	23	5	14	0	9	1
TPR	1.00	1.00	1.00	0.94	0.54	0.25	0.48	0.00	0.67	0.08

**Table 2:** Detection metrics for three different pre-processing techniques used before training a convolutional neural network: standard spectrograms, mel-scale frequency banks, and mel-frequency cepstrum coefficients. Also included for comparison is the same metrics for the HMM. The accuracies were based on the detectors’ ability to discern between background noise and gunshots up to 800 m away.

	TPR	FPR	FP per Hour	Precision	Recall	*F*${}_{1}$ Score
Spectrograms	0.86	0.13	228.0	0.85	0.86	0.85
Freq Banks	0.80	0.04	80.7	0.97	0.80	0.88
MFCC	0.78	0.10	185.8	0.86	0.78	0.82
HMM	0.64	0.08	145.0	0.92	0.64	0.75
